# News and Notes

**Published:** 1901-11

**Authors:** 


### *NEWS AND NOTES.*

---

THE *Atlanta Medical and Surgical Journal* and *Southern Medical Record* having been consolidated into the ATLANTA JOURNAL-RECORD OF MEDICINE, exchanges will please be sent only to the latter. We are getting many duplicates.

---

DR. FLOYD MCRAE has returned from a three months' trip to Europe.

---

DR. WM. PERRIN NICOLSON has returned from a two weeks' trip to the Pan-American Exposition and New York.

---

THE State Medical Board met in annual session at Atlanta, October 9, and examined 25 applicants for license to practice medicine.

---

DR. PARK HOWELL, who has been doing duty as acting assistant surgeon at Fort Duchesne, Utah, has recently passed successfully the examination for assistant surgeon in the regular army.

---

TEN THOUSAND DOLLARS will be distributed by the Porto Rico Relief Association of New York City among the various hospitals of Porto Rico that suffered from the great fire on that island two years ago.

---

ON October 5, under the auspices of the Chicago Medical Society, a banquet was given in honor of Nathan Smith Davis, M.D., LL.D., who is the oldest living president of that society. The banquet took place in the Auditorium Hotel, Chicago.

THE first patent medicine, it is asserted by an exchange, ever put up for sale in America was a proposed cure for tuberculosis. It was called "Tuscarora Rice," and was compounded and sold by a Mrs. Masters, who erected a large establishment for its manufacture in New Jersey about 1711.

---

PRIVY COUNCILOR VINCENZ CZERNY, professor of surgery of the University of Heidelberg for the past twenty-seven years, and one of the most eminent authorities on surgery in the world, is in San Francisco on a visit. Professor Czerny will later visit the cancer hospitals of New York with a view of inspecting them.

---

DR. I. SAYLIN, a Buffalo physician, was arrested a few days ago apparently on suspicion of complicity in some anarchistic plot. It was alleged that he and Emma Goldman and others met in Buffalo to arrange the details of the plot to assassinate the President. He was only held for a short time, and then released, the evidence not being sufficient to hold him.

---

DR. WILLIAM A. WARFIELD, first assistant surgeon of the Freedmen's Hospital, Washington, D. C., has been appointed surgeon-in-chief, vice Dr. A. M. Curtis, whose resignation was accepted last August. Dr. Warfield has been connected with the institution since October, 1894, at which time he entered the hospital as an interne. The salary of the present position is $3,000 a year. An assistant to Dr. Warfield will be selected by a civil service examination to be held October 29th and 30th.

---

CHICAGO's health department calls attention to the fact that there is an epidemic of typhoid fever all over the United States. In Chicago the record shows that the typhoid mortality for the past three months is four times what it was during the corresponding quarter of last year. In New York the hospitals are crowded with typhoid patients, although the prevalence of the disease there may be due to the general tearing up of the streets. In Boston, Baltimore, Cincinnati, Minneapolis, New Orleans, Philadelphia, Pittsburg, St. Louis and Washington more than the usual autumnal increase is reported.

---

ONE of the striking features of the exhibit of the Department of Agriculture at Buffalo, says *American Medicine*, is a collection of silks dyed with food adulterants. One piece of silk is a brilliant red from a substance called "rosaline," used for coloring meats, such as corned beef and sausage. A yard of pink is tinted with dye from preserved cherries and another yard of salmon hue owes its beauty to currant jam. Various kinds of jellies give other colors; there is a fine purple from port wine, a magenta from Burgundy, a light red from tomato catsup, and a pretty yellow from soda-water flavoring.

---

THE Rio Chemical Co., manufacturers of "Celerina," "Aletris Cordial" (Rio), and S. H. Kennedy's "Ext. Pinus Canadensis" (white and dark) have moved to New York and will have no office at St. Louis or any place in the United States except New York. Their address in New York City is 56 Thomas street. Their reasons for moving were that their business had grown to such proportions that they desired to be where they could have better facilities for procuring the various ingredients that enter into the composition of the goods they manufacture. They say that their foreign business has grown to such proportions that there is scarcely a country in the world where educated physicians do not use their goods.

---

**This Journal and Atlanta Semi-weekly Journal one year for $1.00 cash in advance. No checks. Strictly to new subscribers.**

AT a recent meeting of the American Association for the Advancement of Sciences the following officers were elected: President, Prof. Asaph Hall, U. S. N., retired, professor of astronomy at Harvard University; Permanent Secretary, L. O. Howard, chief entomologist, Agricultural Department, Washington; Assistant Permanent Secretary, Richard Clifton, Agricultural Department, Washington; General Secretary, D. T. McDougal, Director of the Laboratories, New York Botanical Gardens; Secretary of Council, Prof. H. B. Ward, of the University of Nebraska; Treasurer, Prof. R. S. Woodward, Columbia University, New York City. The next meeting will be held at Pittsburg, June 28 to July 3, 1902.

---

THE report of the Surgeon-General of the Army was issued on October 8th. It contains the customary financial statement and deals with the following subjects: Artificial limbs and their commutation, appliances, trusses, etc.; Providence Hospital, Army and Navy General Hospital, Hot Springs, Ark., and the Army General Hospital for Tuberculosis, Fort Bayard, N. M.; the army medical museum and the library of the surgeon-general's office; medical officers of the army and volunteers, contract surgeons, and dental surgeons; the hospital corps and army nurse corps; medical and hospital supplies; medical inspections; recruiting, and the identification of deserters and other undesirable men. The health of the army, the prevalence of special diseases, and injuries, receive exhaustive notice. Finally, the boards for the study of the ætiology and prevention of yellow fever and for the investigation of tropical diseases in the Philippines, as also the exhibit of the medical department at the Pan-American Exposition, are dealt with. An abstract of the report will be published later.

THE Comptroller of the Treasury has decided that under the Act of March 3, 1901, contract surgeons are entitled to reimbursement of actual expenses on journeys performed from April 21, 1898, to July 6, 1898, and to mileage as in case of officers of the army after July 6, 1898, and to salaries when on ordinary or sick leave from April 21, 1898, to March 3, 1901, as in the case of officers of the army. Heretofore it has been held that their contracts did not entitle them to the allowances in question. The traveling expenses and mileage are to be computed from the place of original acceptance of offer of employment to place of entry on duty.

---

THE Vth International Congress of Criminal Anthropology in its meeting at Amsterdam, Sept. 9–14, 1901, passed the following resolution:

"The members of the Vth International Congress of Criminal Anthropology are in favor of the establishment of Psycho-Physical Laboratories for the practical application of physiological psychology to sociological and abnormal or pathological data, especially as found in institutions for the criminal, pauper and defective classes and in hospitals and also as may be observed in schools and other institutions."

This Congress consists of distinguished specialists from all over Europe, and it is the highest authority. In our country up to date, the following associations have passed the same resolution but referred it to the Department of the Interior: *Four National Medical Societies Associations:* The Amer. Med. Assoc'n., the Association of American Med. Editors, American Medico-Psychological Asssociations and the Association for the Study and Cure of Inebriety. *Thirteen State Medical Societies:* Connecticut, Indiana, Kansas, Kentucky, Louisiana, Minnesota, Mississippi Valley Med. Association, N. Dakota, New Jersey, Pennsylvania, Texas and Wisconsin. *Three City Medical Societies:* St. Louis, Chicago and Syracuse.

AN INTERNATIONAL HEALTH SERVICE.—One of the questions which, it is said, will receive attention at the coming sessions of the International Conference of American States, to be held in the City of Mexico this month, is the establishment of an international health service. Some time ago the American delegates to the Conference requested Surgeon-General Wyman, of the Marine Hospital Service, to submit suggestions, and he sent to ex-Senator Henry G. Davis, chairman of the United States delegation, a provisional plan entitled "International Sanitation—Pan-American Republics." The plan contemplates an international agreement on measures for the elimination of yellow fever from seaport cities or towns which are or have been endemic habitats of the disease. Surgeon-General Wyman proposes the appointment of an international sanitary commission, to consist of five members, no two of whom are to be residents or citizens of the same republic, the duty of which shall be to visit infected towns or seaports and report on needed sanitary measures. The report is filed with the president of the republic containing the infected port and with the Bureau of American Republics. It then becomes the duty of the authorities to put into force the measures recommended. To insure execution of these measures, Surgeon-General Wyman proposes the imposition of discriminating tonnage dues on vessels arriving from infected ports where sanitary projects are not completed within one year from date of report by the commission.

---

MEDICAL SOCIETY OF THE MISSOURI VALLEY.—The fourteenth annual session of this society convened in St. Joseph on Thursday, September 19th, President Treynor in the chair. After passing resolutions on the death of the President, the society adjourned to allow its members to attend the McKinley memorial services. On Thursday evening the society boarded a special train of Pullman sleepers for Eureka Springs, Arkansas, where the annual outing occurred, and the regular program carried over from St. Joseph was presented: Address of Welcome, Hon. W. M. Brown, Mayor of Eureka Springs; Address on Behalf Local Profession, Dr. J. B. Bolton; Response on Behalf of the M. S. M. V., Dr. V. L. Treynor, President; Dr. Palmer Findley, Chicago, "An Exhibition of Specimens, Illustrating the Cause of Uterine Hemorrhage"; Dr. E. S. Pettyjohn, Chicago, "The Unreliability of Children's Testimony;" Dr. S. Grover Burnett, Kansas City, "Effects of 190° F. Temperature on Man; the Cell Lesion; A Case"; Dr. H. D. Jerowitz, Kansas City, "Scarlet Fever"; Dr. Chas. E. Davis, Eureka Springs, "Some Twentieth Century Thoughts in Medicine"; Dr. William Jepson, Sioux City, "It is Rational to Operate upon Every Case of Appendicitis as Soon as Recognized"; Dr. LeRoy Crummer, Omaha, "The Use of Gartner's Tonometer, with Demonstration of the Instrument"; Dr. Charles Geiger, St. Joseph, "Syphilis"; Dr. P. I. Leonard, St. Joseph, "Some Aspects of Syphilis." On Friday evening a reception and ball was tendered the visitors by the local profession and citizens of Eureka Springs, and Saturday was devoted to sight-seeing in the mountains, several tallyho coaches and sixty saddle horses being provided for the purpose. After a sumptuous dinner on Saturday evening the members left for the return trip, arriving in St. Joseph on Sunday morning. Following is a list of officers elected for the year: President, R. E. Moore, Omaha; First Vice-President, A. D. Wilkinson, Lincoln; Second Vice-President, M. F. Weymann, St. Joseph; Treasurer, Donald Macrae, Council Bluffs; Secretary, Charles Wood Fassett, St. Joseph. Semi-annual meeting will be held in Lincoln in March, 1902.

---

The following is the preliminary program of the Fourteenth Annual Session of the Southern Surgical and Gynecological Association, which will convene at Richmond, Virginia, November 12, 13, 14, 1901:

1. The President's Address. Dr. Manning Simons, Charleston, S. C.
2. Laceration of the Cervix, and its Consequences. Dr. E. S. Lewis, New Orleans, La.
3. Repair of the Complete Laceration of the Perineum in a Girl of Nine Years, Produced by the Finger of the Obstetrician at the Patient's Birth. Dr. H. A. Royster, Raleigh, N. C.
4. Vaginal Puncture or Incision: An Unsurgical Procedure. Dr. Joseph Price, Philadelphia, Pa.
5. Pelvic Hematocele and Hematoma. W. P. Manton, Detroit, Mich.
6. Paper. Dr. Floyd W. McRae, Atlanta, Ga.
7. Retrodisplacement of the Pregnant Uterus: its Surgical Treatment. Dr. Wm. A. Quinn, Henderson, Ky.
8. Treatment of Pelvic and Abdominal Tumors Complicating Pregnancy: With report of Cases. Dr. Rufus B. Hall, Cincinnati, Ohio.
9. Puerperal Septicemia. Dr. Wm. R. Pryor, New York, N. Y.
10. A Unique case of Extra-Uterine Pregnancy, with presentation of Specimen. Dr. H. Tuholske, St. Louis, Mo.
11. Surgical Treatment of the Dysmenorrhea in Unmarried Women and Girls. Dr. J. T. Wilson, Sherman, Texas.
12. The Surgical Treatment of Dysmenorrhea. Dr. Henry D. Fry, Washington, D. C.
13. Paper. Dr. W. L. Robinson, Danville, Va.
14. Tuberculosis of the Female Generative Organs. Dr. J. B. Murphy, Chicago, Ills.
15. Cancer of the Female Urethra, with Report of Cases. Dr. C. Jeff. Miller, New Orleans, La.

16. The Angiotribe. Its Use and Abuse. Dr. Jas. N. Ellis, Atlanta, Ga.
17. Some Points in the Treatment of Appendicitis. Dr. A. M. Cartledge, Louisville, Ky.
18. Some of the Avoidable Causes for Disaster in Appendicitis Work. Dr. Robert T. Morris, New York, N.Y.
19. Gunshot Wounds of the Abdomen. Dr. John C. Wysor, Clifton Forge, Va.
20. Gunshot Wounds of the Abdomen. Dr. Wallace Neff, Washington, D. C.
21. Penetrating Wounds of the Abdomen, with Histories of Six Successful Laparotomies and Statistical Tables of 152 Abdominal Sections done at Charity Hospital, in New Orleans, La. Dr. E. D. Fenner, New Orleans, La.
22. Closure of the Abdominal Incision. Dr. I. S. Stone, Washington, D. C.
23. Paper. Dr. Felix A. Larue, New Orleans, La.
24. (a) The Results Obtained in Sixty Operations for Prostatic Hypertrophy: Demonstrations of a New Combined Cautery Incisor. (b) Catheterization of the Ureters in the Male, with Report of Cases. Dr. Hugh H. Young, Baltimore, Md.
25. Perineal Prostatectomy. Dr. Alex. Hugh Ferguson, Chicago, Ills.
26. Report of a Case of Gangrene of the Gall Bladder. Dr. Geo. Ben Johnson, Richmond, Va.
27. Report of Cases of Gall Stones. Dr. J. A. Goggans, Alexander City, Ala.
28. Gastrostomy in the Treatment of Impermeable Stricture of the Æsophagus. Dr. Hugh M. Taylor, Richmond, Va.
29. Æsophagotomy, with Report of Nine Successful Operations on an Infant Forty-six Days Old, with Illustrations. Dr. John W. Long, Salisbury, N. C.

30. Two cases of Nephro-Ureterectomy, with Remarks. Dr. J. Wesley Bovee, Washington, D. C.
31. The Treatment of Procidentia Uteri. Dr. Chas. P. Noble, Philadelphia, Pa.
32. Clinical Report of Two Cases of Osteo-Sarcoma of the Maxilla, Treated by Excision, with Statement of Condition of Patient After One Year. Dr. Hermann B. Gessner, New Orleans, La.
33. (a) Spiral Section of Fibroid Tumors in Lower Portion of Uterus to Facilitate their Removal. (b) Puncture Scissors and Counter-Pressure Instrument for Vaginal Puncture in Vaginal Drainage. (c) The use of Adhesive Plaster for Prevention of Laceration of the Perineum in Forcep Delivery. Dr. Geo. H. Noble, Atlanta, Georgia.
34. Operations on the Liver. Dr. W. E. B. Davis, Birmingham, Ala.
35. Hepatic Drainage. Dr. John B. Deaver, Philadelphia, Pa.
36. Report of Two Interesting Cases. Dr. Jos. Taber Johnson, Washington, D. C.
37. Tubercular Peritonitis. Dr. Samuel Lloyd, New York, N. Y.
38. Paper. Dr. M. C. McGannon, Nashville, Tenn.
39. Hodgen's Splint for the Treatment of Fracture of the Thigh. Dr. Geo. S. Brown, Birmingham, Ala.
40. A Peculiar Succession of Septic Wounds, Occurring in and around Alexander City, Ala., with Report of Cases. Dr. A. J. Coley, Alexander City, Ala.
41. Pott's Disease and Some of its Complications. Dr. A. R. Shands, Washington, D. C.
42. Surgery of the Pancreas. Dr. W. D. Haggard, Jr., Nashville, Tenn.

